# Granzyme B+ B cells detected by single-cell sequencing are associated with prognosis in patients with intrahepatic cholangiocarcinoma following liver transplantation

**DOI:** 10.1007/s00262-023-03609-x

**Published:** 2024-02-22

**Authors:** Ji-Qiao Zhu, Ying Zhu, Man Qi, Ye Zeng, Zhen-Jia Liu, Cheng Ding, Tao Zhang, Xian-Liang Li, Dong-Dong Han, Qiang He

**Affiliations:** 1 Department of Hepatobiliary and Pancreaticosplenic Surgery, Beijing Organ Transplant Center, Beijing Chaoyang Hospital, Capital Medical University, No. 8 Gongtinan Road, Chaoyang District, Beijing, 100020 People’s Republic of China; 2 Department of Clinical Psychology, Mental Hospital of Jianqu Administration Bureau of Jiangsu Province, Nanjing, 210031 Jiangsu People’s Republic of China; 3 Pathology Department, Beijing Chaoyang Hospital, Capital Medical University, Beijing, 100020 People’s Republic of China; 4 Clinical Lab, Tongji Medical College, Wuhan Children’s Hospital (Wuhan Maternal and Child Healthcare Hospital), Huazhong University of Science & Technology, Wuhan, 430070 Hubei People’s Republic of China; 5 Department of Infectious Diseases and Clinical Microbiology, Beijing Chaoyang Hospital, Capital Medical University, Beijing, 100020 People’s Republic of China; 6 Department of Hepatobiliary Surgery, China-Japan Friendship Hospital, No. 2 Yinghua East Street, Chaoyang District, Beijing, 100029 People’s Republic of China

**Keywords:** Granzyme B+ B cells, Intrahepatic cholangiocarcinoma, Liver transplantation, Single-cell sequencing, Immune escape

## Abstract

B cells possess anti-tumor functions mediated by granzyme B, in addition to their role in antigen presentation and antibody production. However, the variations in granzyme B+ B cells between tumor and non-tumor tissues have been largely unexplored. Therefore, we integrated 25 samples from the Gene Expression Omnibus database and analyzed the tumor immune microenvironment. The findings uncovered significant inter- and intra-tumoral heterogeneity. Notably, single-cell data showed higher proportions of granzyme B+ B cells in tumor samples compared to control samples, and these levels were positively associated with disease-free survival. The elevated levels of granzyme B+ B cells in tumor samples resulted from tumor cell chemotaxis through the MIF- (CD74 + CXCR4) signaling pathway. Furthermore, the anti-tumor function of granzyme B+ B cells in tumor samples was adversely affected, potentially providing an explanation for tumor progression. These findings regarding granzyme B+ B cells were further validated in an independent clinic cohort of 40 liver transplant recipients with intrahepatic cholangiocarcinoma. Our study unveils an interaction between granzyme B+ B cells and intrahepatic cholangiocarcinoma, opening up potential avenues for the development of novel therapeutic strategies against this disease.

**Supplementary Information:**

The online version contains supplementary material available at 10.1007/s00262-023-03609-x.

## Introduction

B cells are commonly recognized as antigen-presenting cells and producers of high-affinity antibodies. Recent studies have reported that B cells can secret granzyme B (GrB) when stimulated by a combination of interleukin-21 (IL-21) and the B cell receptor (BCR) [1–4]. Chesneau et al. demonstrated that B cells from tolerant renal transplant recipients were able to inhibit the proliferation of effector T cells in a GrB-dependent manner upon activation [5]. Xu et al. further revealed that GrB+ B cells acted as a feedback loop for effector T cells in patients experiencing acute rejection following liver transplantation [6]. These studies suggest that GrB+ B cells can contribute to re-establishing immune homeostasis and maintaining tolerance by suppressing immune responses. Additionally, GrB+ B cells have been found to play a significant role in cancer immunosurveillance and are associated with a better prognosis for cancer patients, indicating their tumor-killing potential [7, 8].

Hepatocellular carcinoma (HCC) and intrahepatic cholangiocarcinoma (iCCA) are etiologically and biologically heterogeneous and have different outcomes, despite being the two most common subtypes of primary liver cancer [9]. A systematic literature search revealed that a 5-year overall survival rate was 47.5% for patients with HCC compared to 30.3% for patients with iCCA after resection [10]. For patients with unresectable iCCA, palliative chemotherapy has been the main regimen [11], resulting in a reported median survival of only 10.6 months [12]. The diverse landscapes of the tumor microenvironment may contribute to therapeutic failures and adverse outcomes [13]. Hence, investigating tumor-infiltrating immune cells is necessary to develop promising therapeutic approaches [14].

Our previous findings highlighted the critical role of GrB+ B cells from patients with HCC in preventing cancer progression following liver transplantation [15]. However, the alterations in GrB+ B cells between tumor and non-tumor tissues have never been elucidated, potentially facilitating immune evasion for tumor cells. In this study, we dissected the tumor immune microenvironment, particularly focusing on GrB+ B cells at a single-cell resolution by integrating iCCA and control samples. We observed elevated levels of dysfunctional GrB+ B cells in the tumor samples compared to control samples. Moreover, we analyzed potential mechanisms and validated the impact of GrB+ B cells on patients’ outcomes following liver transplantation. Our study sheds light on the tumor progression, invasiveness, and the development of novel therapeutic strategies against iCCA, benefiting a wider range of patients.

## Results

### Single-cell analysis of microenvironment in control and tumor samples

**Fig. 1:**
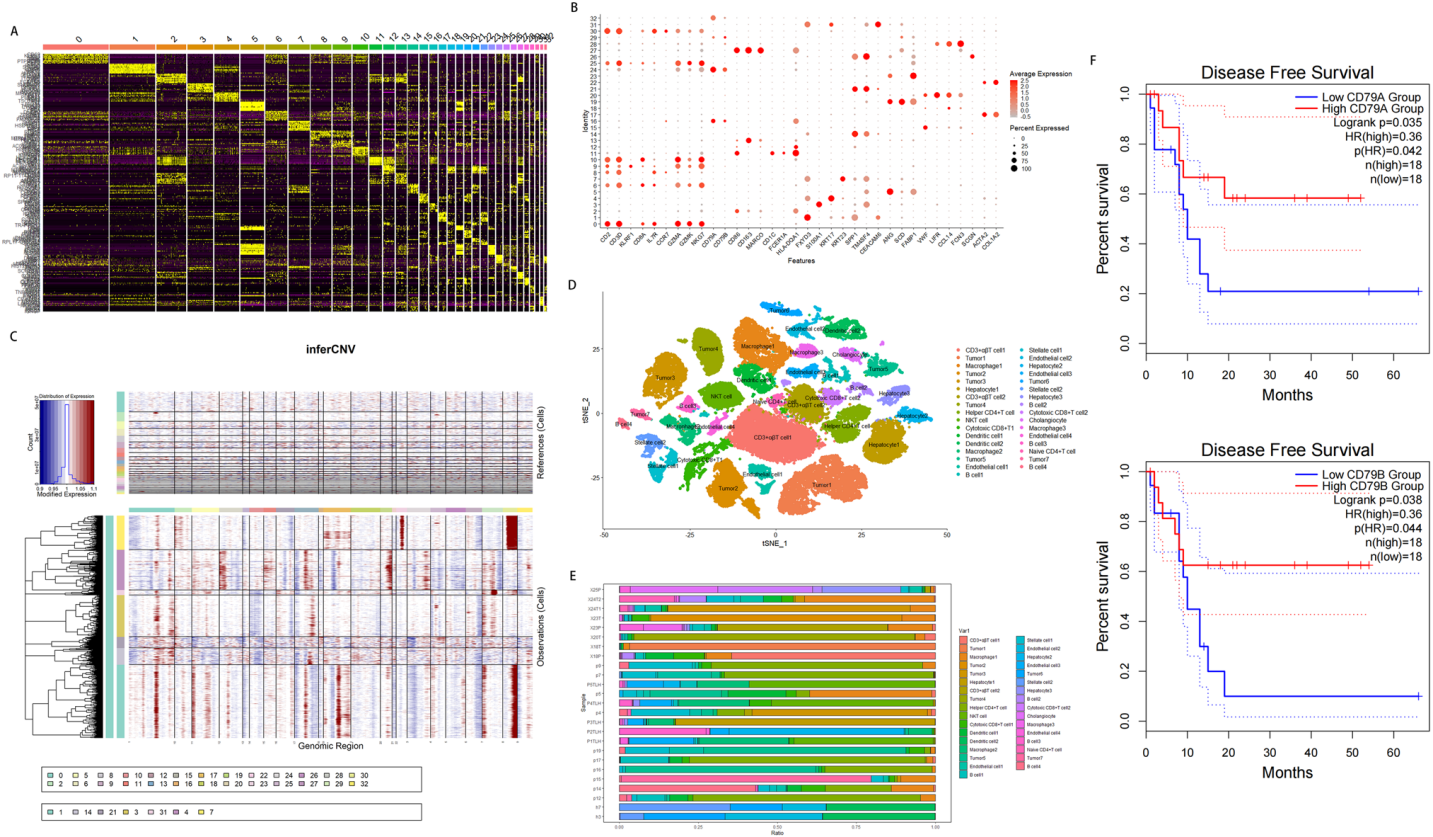
Overview of transcriptome data from iCCA and control samples, highlighting the impact of B cells on DFS. **A** A heatmap showing the expression levels of specific markers in each cluster. **B** A dot plot displaying the expressions of representative well-known markers in each cluster. **C** Chromosomal landscape of inferred large-scale copy number variations distinguishing malignant cells from non-malignant cells. **D** The t-distributed stochastic neighbor embedding plot demonstrating the main cell types in hepatic samples. **E** Proportions of the main cell types in each hepatic sample. **F** Kaplan–Meier curves for disease-free survival (DFS) in the TCGA CHOL data stratified according to high versus low CD79A (upper) and CD79B (lower) expression, respectively

We conducted a search in public repositories for single-cell transcriptome profiles from patients with and without iCCA as detailed in Materials and Methods. After exclusion, we obtained four datasets from the GEO database [16–19]. In our study, we analyzed single-cell RNA-seq data from a total of 22 patients, including five patients without iCCA who provided five control samples, and 17 patients with iCCA who provided both three control samples and 17 tumor samples (Fig. S1A). After applying quality control and normalization steps, we obtained a combined total of 44,748 cells. Of these, 22,314 cells came from the control samples and 22,434 cells from the tumor samples (Fig. S1B). Subsequently, we identified 33 distinct clusters of cells (Fig. S1C) and determined the cell type of each cluster by examining cluster-specific genes (Fig. 1A, B). For instance, we used CD79A and CD79B to identify the B cell cluster. Additionally, we distinguished malignant cells from non-malignant cells by inferring large-scale chromosomal CNVs. Non-malignant cells from each tumor sample were identified if they were predominantly present in clusters containing cells from the control samples. On the other hand, cells displaying with whole-chromosome deletions or amplifications from the tumor samples were categorized as malignant cells. We calculated and compared CNV scores across different origins, groups, and clusters (Fig. S1D, F). Ultimately, we identified a total of 13,206 cells belonging to seven clusters as iCCA cells (Fig. 1C). Furthermore, we annotated the following cell lineages based on literature references and the CellMarker database [16–19]: CD3^+^αβT cells, Macrophages, Hepatocytes, Helper CD4^+^T cells, NKT cells, Cytotoxic CD8^+^T cells, Dendritic cells, Endothelial cells, B cells, Stellate cells, Cholangiocytes, Naive CD4^+^T cells (Fig. 1D). We calculated the proportions of all cell types in each sample (Fig. 1E), revealing variation in cell composition among samples. Additionally, we compared the distribution of B cells (based on CD79A and CD79B expressions) between the control and tumor groups. Our analysis showed that the proportions of B cells, either individually (in four clusters) or collectively, were similar between the two groups (Fig. S1G, H). Lastly, we assessed the effect of B cells on tumor prognosis using TCGA data. We utilized the ‘Survival analysis’ module of the GEPIA2 tool to compare the DFS based on the expression levels of CD79A and CD79B in iCCA patients. As depicted in Fig. 1F, low CD79A and CD79B expressions, below the median, were significantly associated with a poor prognosis of DFS (*p* < 0.05). In conclusion, our comprehensive analysis of 44,748 cells from 22 patients reveals significant inter-tumoral and intra-tumoral heterogeneity, with B cells exhibiting a strong influence on tumor recurrence.

### Increased proportions of GrB+ B cells in tumor samples are associated with patients’ survival

**Fig. 2:**
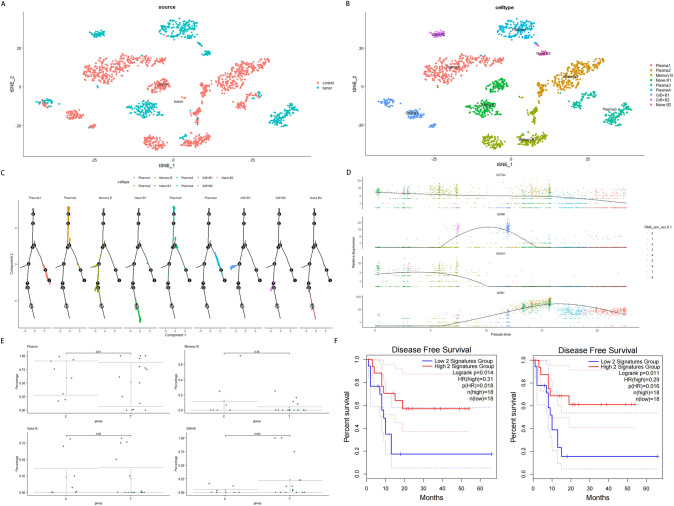
Characterization of GrB+ B cells identified in B cell clusters. **A** The t-distributed stochastic neighbor embedding (t-SNE) plot demonstrating the distribution of malignant and non-malignant cells in B cell clusters based on CD79A and CD79B expression. **B** The t-SNE plot showing the main subtypes in B cell clusters. **C** Distribution plots of each B cell subcluster in pseudo-sequential trajectories. **D** Gene expression variations with pseudo-time. **E** Comparison of proportions of B cell subtypes between the control and tumor groups. **F** Kaplan–Meier curves for disease-free survival in TCGA CHOL data stratified by high vs. low expression of a combination of MS4A1 and GZMB (left) and MZB1 and GZMB (right), respectively

Our subsequent focus was on the clusters characterized by CD79A and CD79B expressions. In our study, we detected 1,993 cells with CD79A and CD79B expressions, consisting of 1,305 cells from the control samples and 688 cells from the tumor samples (Fig. 2A). We observed varying numbers of B cells identified in each sample, ranging from single cells to 594 cells (Fig. S2A). These cells clustered into nine distinct subsets (Fig. S2B). To define B cell subsets, we utilized web-based cell annotation resources to identify cluster-specific genes (Fig. S2C). Based on this analysis, memory B cells expressing MS4A1 and CD27 were annotated to cluster 2, naïve B cells expressing MS4A1, IGHD, and IGHM to clusters 3 and 8, plasma cells expressing MZB1 to clusters 0, 1, 4, and 5, and GrB+ B cells expressing GZMB to clusters 6 and 7 (Fig. S2D, Fig. 2B), in line with their known characteristics [20, 21]. As GrB+ B cells are a type of regulatory cells, we analyzed the B cell subsets to infer their developmental trajectory and assess state divergence. Our results showed that naïve B cells, memory B cells, and plasma cells were located at the ends of the trajectory, whereas GrB+ B cells were enriched in the middle (Fig. S2E, Fig. 2C), suggesting that GrB+ B cells resided in a transitional state [22, 23]. We selected GZMB, MS4A1, CD79A, and MZB1 to investigate their expressions over pseudo-time (Fig. 2D). In contrast to the other three genes, GZMB expression was only elevated at the intermediate stage, as confirmed by the heatmap analysis (Fig. S2F), further buttressing the transitional role of GrB+ B cells. After that, we compared the proportions of B cell subsets between the two groups. Samples from p5 and p16 exclusively provided GrB+ B cells, while samples from p15, p19, and X18T contained only plasma cells (Fig. S2G). Tumor samples exhibited higher proportions of GrB+ B cells compared to control samples, although the difference did not reach significance (Fig. 2E). Moreover, we investigated the effect of GrB+ B cells on tumor prognosis. We used combinations of MS4A1 and GZMB, and MZB1 and GZMB as surrogates for GrB+ B cells in TCGA data, considering their transitional nature, respectively (Fig. 2F). Both gene signatures, when higher than the median, were significantly associated with a longer time to recurrence in patients with iCCA (*p* < 0.05). These findings suggest that the percentages of GrB+ B cells are elevated in tumor samples and associated with DFS.

### Enriched GrB+ B cells in iCCA issues result from chemotaxis of tumor cells

**Fig. 3:**
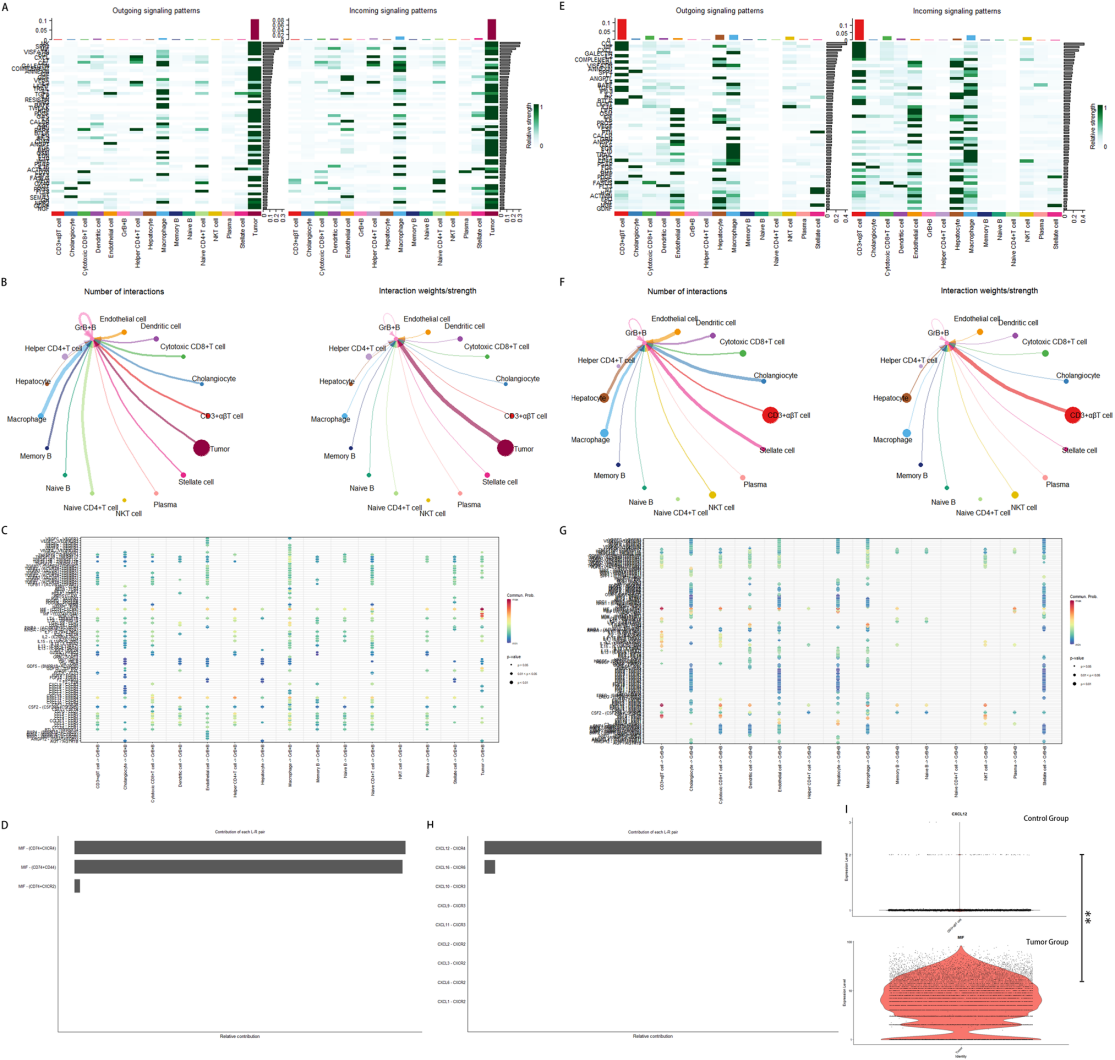
Enriched GrB+ B cells in patients with iCCA result from chemotaxis of tumor cells. **A** Heatmaps showing the outgoing and incoming signaling patterns in tumor samples, respectively. Darker color implies a higher enrichment of the signaling pathway in the corresponding cell type. **B** Circle plots showing the number of interactions and interaction weights/strength between GrB+ B cells (acting as a receptor) and other cells (serving as ligands) in tumor samples. Dot size indicates the quantity of each cell type. Each line color indicates the ligands expressed by the cell population represented in the same color (labeled). The lines connect to the cell types that express the cognate receptors. The line thickness is proportional to the number of L-R pairs. **C** A dot plot displaying the L-R pairs in tumor samples, which contribute to the signaling from ligand cells to GrB+ B cells. Dot color reflects communication probabilities and dot size represents computed *P*-values. Empty space means zero communication probability. **D** Relative contribution of each L-R pair to the overall MIF signaling network. **E** Heatmaps showing the outgoing and incoming signaling patterns in control samples, respectively. **F** Circle plots showing the number of interactions and interaction weights/strength between GrB+ B cells (acting as a receptor) and other cells (serving as ligands) in control samples. **G** A dot plot displaying the L-R pairs in control samples with GrB+ B cells acting as a receptor. **H** Relative contribution of each L-R pair to the overall CXCL signaling network. **I** Violin plots showing the expression distribution of MIF in tumor cells from the tumor samples and CXCL12 in CD3.^+^αβT cells from the control samples involved in the corresponding signaling network, respectively, with comparison of log-normalized gene expression values. ***P = 2.200384e − 17

The migration of B cells from the bloodstream and lymph to the liver is regulated by adhesion molecules and chemokine receptors [24]. We utilized the ‘CellChat’ package to infer and compare cell–cell communication based on the single-cell profiles. We initially presented an overview of the signaling pathways in all cell types in tumor samples (Fig. 3A). The various signaling pathways exhibited different levels of interaction strength between the cell types. Tumor cells exhibited the highest relative strength in both incoming and outgoing signaling pathways, with the MK signaling pathway ranking first. Then, we analyzed the interactions between GrB+ B cells and other cell types. GrB+ B cells, serving as targets or sources, engaged in numerous interactions with other cell types, with the strongest interaction strength observed with tumor cells (Fig. S3A, Fig. 3B). In the subsequent analysis of the potential pathways between GrB+ B cells and tumor cells, the most important incoming and outgoing L-R pairs were the signaling complexes, macrophage migration inhibitory factor (MIF) -(CD74 + CXCR4) and lymphotoxin-alpha (LTA)–(LTB + LTBR), respectively (Fig. S3B, C, Fig. 3C, D). Conversely, CD3^+^αβT cells replacing tumor cells exhibited the highest relative strength in both incoming and outgoing signaling pathways in control samples (Fig. 3E), with which GrB+ B cells interacted most prominently as targets or sources (Fig. S3D, Fig. 3F). Upon analysis of the incoming and outgoing communication patterns, CXCL12-CXCR4 chemokine signaling emerged as the most important L-R pair between GrB+ B cells and CD3^+^αβT cells (Fig. S3E, Fig. 3G, H). Gene abundance comparison between the MIF and CXCL pathways revealed significantly higher MIF expression in tumor cells than CXCL12 expression in CD3^+^αβT cells (Fig. 3I). Furthermore, the expressions of CD74 and CXCR4 were higher in GrB+ B cells from the tumor group compared to the control group (Fig. S3F). Lastly, MKI67 expression in GrB+ B cells was analyzed to assess the impact of clonal expansion in tumor samples (Fig. S3F). MKI67 expression was found to be significantly reduced in both groups. Taken together, these findings highlight the influence of tumor cells chemotaxis on GrB+ B cells through the MIF- (CD74 + CXCR4) signaling pathway.

### Impaired anti-tumor function of GrB+ B cells in tumor samples

**Fig. 4:**
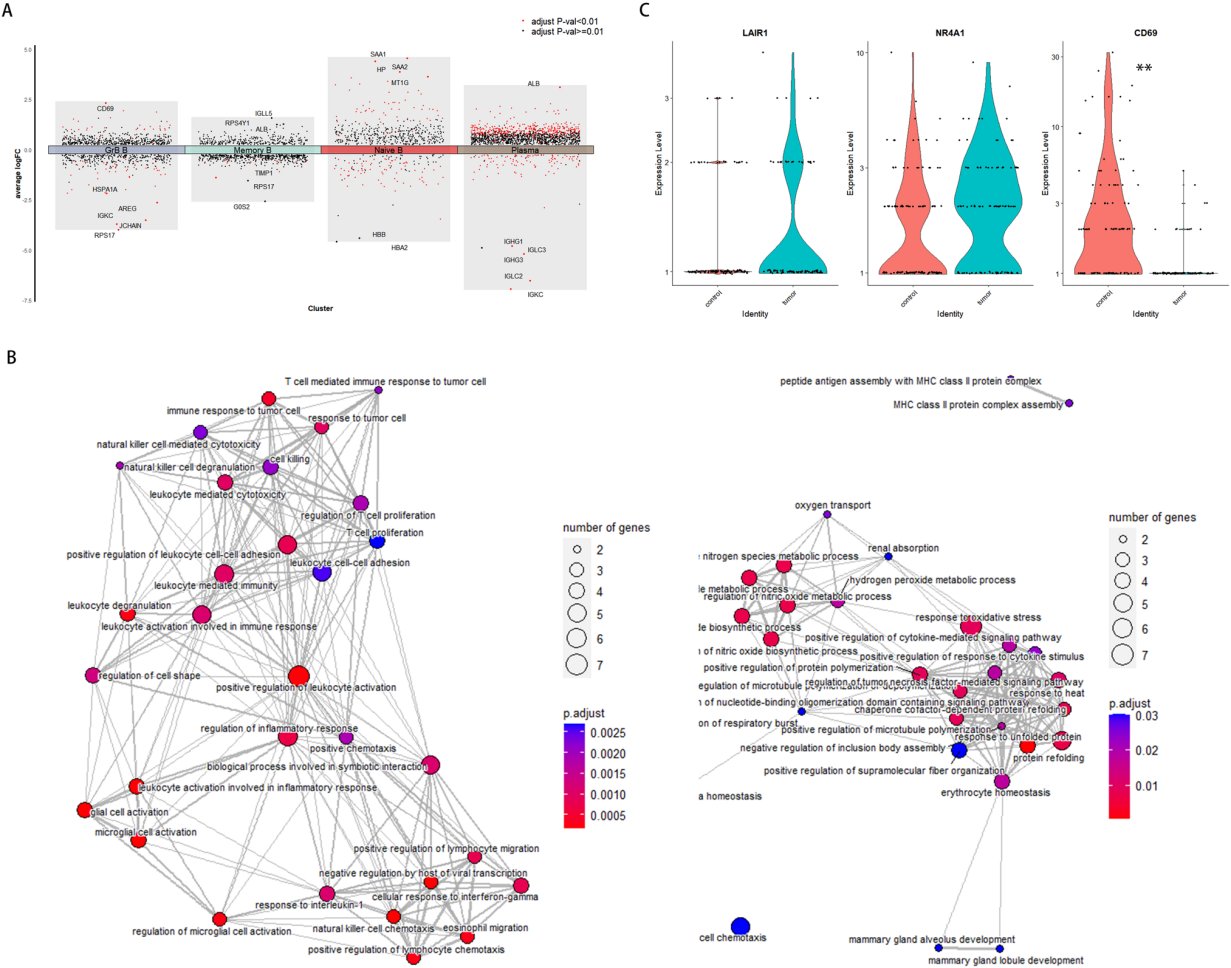
The changes in the functional states of GrB+ B cells between the tumor and control tissues. **A** Differentially expressed genes (DEGs) of B cell subsets detected between the tumor and control groups. The y-axis indicates the average log_2_FC of gene expression. **B** Top biological pathways enriched for DEGs identified in GrB+ B cells in the tumor samples in comparison with those in the control samples. Dot size represents the numbers of down-regulated (left) and up-regulated (right) genes in each GO term. **C** Violin plots showing the expression distributions of LAIR1, NR4A1, and CD69 in GrB+ B cells from the tumor and control groups, with comparison of log-normalized gene expression values, respectively. LAIR1, *P* = 0.1025; NR4A1, *P* = 0.9655; ***CD69, *P* = 0.0005

Tumor samples exhibited higher proportions of GrB+ B cells, which could theoretically suppress tumor progression through the activity of GrB [25, 26]. Thus, we sought to investigate whether the anti-tumor function of GrB+ B cells was impaired following chemotaxis. To evaluate the functions of GrB+ B cell, we initially identified highly variable features derived from GrB+ B cells in the control and tumor groups. The results revealed that cytoplasmic translation and translation were the top two functional terms shared by GrB+ B cells in both groups (Fig. S4A). Subsequently, regulon activities and specificity scores were calculated separately for each group. The similarity of main regulon activities and specificities further supported the findings of the GO analysis (Fig. S4B, C). Next, The DEGs analysis between the tumor and control samples was conducted (Fig. 4A). The analysis uncovered a down-regulation of immune response-related genes and associated functions, alongside an up-regulation of genes related to cell chemotaxis, consistent with the results of ‘CellChat’ analysis (Fig. 4B). As the anti-BCR and IL-21 are the most potent stimuli for GrB production [1, 2, 4], the expression levels of their receptors were compared. Notably, BCR and IL21R were scarcely detectable in B cells from both groups (Fig. S4D). Moreover, the expression levels of LAIR1 and NR4A1, which were reported to be a kind of exhausted genes, were analyzed as well as CD69 [27, 28]. GrB+ B cells from tumor samples exhibited slightly higher expression levels of LAIR1 and NR4A1 but a lower level of CD69 expression (Fig. 4C). The results suggest that tumor samples contain less mature but slightly exhausted GrB+ B cells, providing a possible explanation for their compromised function. Overall, these findings indicate that the GrB+ B cells in tumor samples are functionally impaired.

### Increased but compromised GrB+ B cells are validated in a clinic cohort

**Fig. 5:**
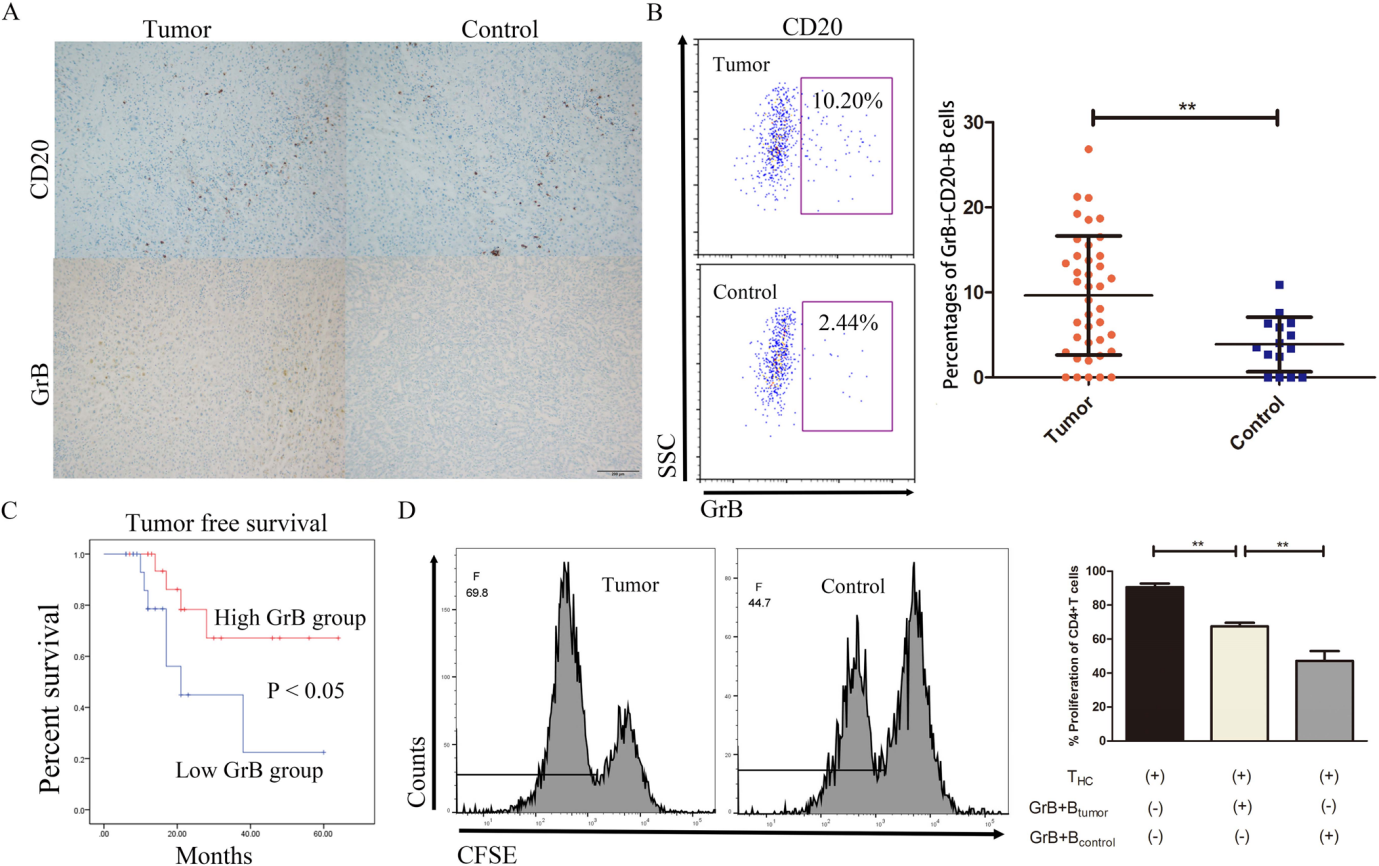
Increased but compromised GrB+ B cells are validated in a clinic cohort. **A** Representative immunohistochemistry images of CD20 and granzyme B (GrB) in iCCA and benign hepatic tissues, respectively. **B** Representative flow cytometry dot plots and comparison of the GrB expression in resting CD20+ B cells from tumor (up, *n* = 40) and control (down, *n* = 15) samples, respectively. **C** Kaplan–Meier curves for tumor-free survival in an independent cohort stratified based on the median GrB expression in LTR with iCCA, as calculated in Fig. 5B. **D** Representative flow cytometry images and comparison of the proliferative responses of CD4^+^T cells co-cultured with activated GrB+ B cells isolated from tumor and control issues (*n* = 3), respectively. CD20+ B cells were stimulated with IgG + IgM (5.4 μg/ml), CpG ODN 2006 (9.6 μg/ml), and IL-21 (50 ng/ml). *iCCA* intrahepatic cholangiocarcinoma, *LTR* liver transplant recipients

In an attempt to validate our findings with respect to GrB+ B cells in tumor samples, we conducted an independent study involving 40 LTR with iCCA and three patients with benign diseases. The characteristics of the LTR are summarized in Table 1. Immunohistochemistry staining demonstrated higher levels of GrB and CD20 in tumor samples compared to control samples (Fig. 5A). To specifically identify GrB+ B cells among other GrB-producing cells, such as T cells and NK cells, we isolated lymphocytes from both tumor samples and control samples followed by multiparametric flow cytometry analysis. The results confirmed a higher percentage of GrB+ B cells in tumor samples compared to control samples (Fig. 5B). After that, the LTR were regrouped based on the median value of the percentage of GrB+ B cells in the tumor group. Notably, the group with a frequency below the median value exhibited higher rates of tumor recurrence rates following liver transplantation (Fig. 5C). We further detected the ligand expression of the signaling pathways. MIF staining was intense in tumor cells, while CXCL12 and CD3 stainings were relatively weak and diffuse in paraffin sections of control samples (Fig. S5A). The expression levels of IgG, IgM, IgE, IgD, IgA, and IL21R on B cells from tumor samples were comparable to those from control samples (Fig. S5B). Previously, we reported that GrB+ B cells from LTR with HCC could directly inhibit the proliferation, migration and invasion of hepatic tumor cells through the action of GrB [15]. In this study, to clarify the changes in their suppressive function, we co-cultured GrB+ B cells from both groups with purified CD4^+^T cells in the presence of anti-CD3/CD28 beads based on the previous results. T cell proliferative responses were stronger in the iCCA group than in the control group upon activation; however, GrB+ B cells from the iCCA samples were still able to suppress T cell proliferation (Fig. 5D). This finding may explain the higher recurrence rates observed in iCCA samples with lower percentages of GrB+ B cells. Furthermore, we employed PCR and Western blot analysis to assess the production of GrB in purified and stimulated B cells, respectively (Fig. S5C). We found that patients with iCCA exhibited lower levels of GrB mRNA upon stimulation in the presence of anti-BCR and IL-21 compared to the control group (left). The protein expression also showed weaker production of GrB in patients with iCCA (right). Consistently, these data suggest an increase in compromised GrB+ B cells in tumor samples, which is associated with patients’ prognosis following liver transplantation.

**Table 1:** Characteristics of patients in the clinic cohort

Parameters	Tumor (*n* = 40)
Age	59.30 ± 11.01
Sex (male)	23
Disease	iCCA
*Maintenance*	
Tacrolimus+Sirolimus	18
Tacrolimus	17
Sirolimus	3
Cyclosporine A	2
Hepatitis	14
Cirrhosis	16
*Differentiation*	
High	3
Middle	27
Low	10
Microvascular invasion	22
Tumor-free survival (month)	21.08 ± 14.95
Cum survival (months)	23.98 ± 15.04

iCCA, intrahepatic cholangiocarcinoma

## Discussion

In our study, tumor cells exploited the chemokines they produce to recruit GrB+ B cells. Consequently, this led to an accumulation of GrB+ B cells in tumor tissues. However, the suppressive function of these GrB+ B cells is compromised, resulting in an impaired anti-tumor immune response. The exact link between tumor cells and compromised GrB+ B cells still required clarification in future research. Our findings offer a potential explanation for the accumulation of GrB+ B cells in tumor tissues and the down-regulation of anti-tumor immune response.

Previous studies have consistently shown that GrB+ B cells possess anti-tumor function, indicating that patients with lower percentages of GrB+ B cells are more susceptible to recurrence or disease progression [8, 15, 21]. Based on this evidence, we hypothesized that the proportion of GrB+ B cells would decrease in tumor tissues compared to non-tumor tissues. However, our analysis of single-cell sequencing datasets obtained from iCCA and control tissues revealed that iCCA tissues exhibited higher percentages of GrB+ B cells. The recruitment of GrB+ B cells to tumor tissues occurs through the MIF-(CD74 + CXCR4) axis. MIF plays a significant role in regulating the recruitment of inflammatory cell, exhibiting chemokine-like functions by interacting with its primary receptor, CD74, and its co-receptor, CXCR4 [29–31]. The binding of MIF to these receptors can promote the migration of B cells via a ZAP-70-dependent pathway [32]. This, in return, affects their proinflammatory activities and sensitivity to Fas-mediated apoptosis [33]. The use of the humanized monoclonal antibody milatuzumab, which targets CD74, can induce slight decreases in proliferation, modifications in migration, and changes in the expression of adhesion molecule in B cells [34].

GrB+ B cells are known for their capacity to induce cytotoxicity in tumor cells [15, 35], resulting in increased activation of caspase-3 and reduced expression of Ki-67 [8]. Currently, the distinction in the function of GrB+ B cells between patients with and without tumors remains unclear. Our study presents novel evidence of the down-regulation of genes associated with the immune response to tumor cells and their functions, suggesting the impairment of GrB+ B cells. We further investigated and validated this impairment through in vitro functional assay in the clinic cohort. The impairment of GrB+ B cells has been correlated with the pathogenesis of various diseases, such as allergic diseases [36], autoimmune diseases [4], and organ transplantation [3, 5]. Despite the accumulation of GrB+ B cells in tumor tissues, their reduced GrB production compared to counterparts in the control group under stimulation reflects the modulation of the anti-tumor immune response following chemotaxis. Moreover, the degradation of the T cell receptor *ζ*-chain by GrB+ B cells within solid tumors may additionally restrict neighboring cytotoxic T cells with anti-tumor activity, thereby promoting cancer progression (37).

## Conclusions

This study offers a comprehensive understanding of GrB+ B cells in patients with iCCA. It reveals a significant association between GrB+ B cells infiltrating tumor tissues and the patients’ prognosis, shedding light on a novel mechanism for tumor immune escape. The findings have the potential to guide the development of therapeutic strategies against tumors.

### Supplementary Information

Below is the link to the electronic supplementary material.

Supplementary file1 (DOC 76536 KB)

## Data Availability

The datasets generated during and/or analyzed during the current study are available from the corresponding author on reasonable request.
