# Supplementary material for: Granzyme B+ B cells detected by single-cell sequencing are associated with prognosis in patients with intrahepatic cholangiocarcinoma following liver transplantation
Source: Cancer Immunol Immunother. 2024 Feb 22;73(3):58. doi: 10.1007/s00262-023-03609-x (PMC10884120; doi:10.1007/s00262-023-03609-x)
Supplement: Supplementary file 1 — Supplementary file1 (DOC 76536 KB) [file 262_2023_3609_MOESM1_ESM.doc]

Supplementary information for

Granzyme B^+^B cells detected by single-cell sequencing are associated with prognosis in patients with intrahepatic cholangiocarcinoma following liver transplantation

Ji-Qiao Zhu^a^*, dr_zhujiqiao@163.com; Ying Zhu^b^*, 842831259@qq.com; Man Qi^c^*, qiman888@163.com; Ye Zeng^d^, cinderella1012@msn.com; Zhen-Jia Liu^e^, 13488725173@163.com; Cheng Ding^a^, dingcheng13@126.com; Tao Zhang^a^, taiyizhangtao@163.com; Xian-Liang Li^a^, [lixianliangbjcy@126.com;](mailto:lixianliangbjcy@126.com;) Dong-Dong Han^f#^, [surghandd@163.com;](mailto:surghandd@163.com;) Qiang He^a#^, [heqiangsurg@163.com](mailto:heqiang@bjcyh.com;)

^a^Department of Hepatobiliary and Pancreaticosplenic Surgery, Beijing Organ Transplant Center, Beijing Chaoyang Hospital, Capital Medical University, Beijing 100020, China

^b^Department of Clinical Psychology, Mental Hospital of Jianqu Administration Bureau of Jiangsu Province, Nanjing, Jiangsu 210031, China

^c^Pathology Department, Beijing Chaoyang Hospital, Capital Medical University, Beijing 100020, China

^d^Clinical Lab, Wuhan Children's Hospital (Wuhan Maternal and Child Healthcare Hospital), Tongji Medical College, Huazhong University of Science & Technology, Wu Han, Hubei 430070, China

^e^Department of Infectious Diseases and Clinical Microbiology, Beijing Chaoyang Hospital, Capital Medical University, Beijing 100020, China

^f^Department of Hepatobiliary Surgery, China-Japan Friendship Hospital, Beijing 100029, China

*These authors contributed equally to this work

^#^**Corresponding authors**: Qiang He and Ji-Qiao Zhu, No. 8 Gongtinan Road, Chaoyang District, Beijing 100020, PR China, Phone +86-(0)-10-85231504, Fax +86-(0)-10-85231503

1. Materials and Methods
2. Supporting figures and figure legends

**Materials and Methods**

*Single-cell RNA-seq data processing*

GSE115469(1), GSE146409(2), GSE138709(3), and GSE125449(4) datasets were downloaded from the gene expression omnibus (GEO) database (https://www.ncbi.nlm.nih.gov/geo/). The GSE115469 dataset included hepatic non-tumor tissues from five patients (P1TLH, P2TLH, P3TLH, P4TLH, and P5TLH). The GSE146409 dataset consisted of hepatic tumor tissues from three patients with colorectal liver metastases, two patients with iCCA, and one patient with a cyst. Only data from patients with iCCA were collected (p1=h3 and p2=h7). The GSE125449 dataset contained set1, which included seven patients with HCC and five patients with iCCA, and set2, which contained two patients with HCC and five patients with iCCA. Again, only data from patients with iCCA were collected (p4, p5, p7, p9, p12, p14, p15, p16, p17, and p19). The GSE138709 dataset included hepatic tumor tissues from four patients with iCCA (X18T, X20T, X23T, X24T1, and X24T2) and hepatic non-tumor tissue from three patients with iCCA (X18P, X23P, and X25P).

Next, the raw digital gene expression matrix was converted to a Seurat object using R (https://www.R-project.org/). Genes that were detected in less than three cells and cells with fewer than 201 genes were filtered out. Additionally, cells with gene overexpression and high mitochondrial content (≥ 3000&5% for GSE115469, ≥ 2000&30% for GSE146409, ≥ 6000&30% for GSE138709, and ≥ 3000&10% for GSE125449) were removed. Afterwards, all the datasets were merged, scaled to unit variance and zero mean. The merged data were then scaled and log-transformed. The top 2000 highly variable genes were identified. ‘CellCycleScoring’ was utilized to assign cell cycle scores for the regression of cell cycle genes. Principal component analysis was applied to reduce dimensionality, with the number of principal components estimated using the Elbow plot. Subsequently, the t-distributed Stochastic Neighbor Embedding (t-SNE) dimensionality reduction was applied to further summarize the principal components. ‘DoubletFinder’ (version 2.0.3) was applied to infer and remove doublets, which involved selecting the top N cells with the highest pANN score(5). The ‘FindAllMarkers’ function was employed to identify differential expression for each cell population, while ‘FindMarkers’ was used for specific populations. Clustering of cells was based on their expression profile with a resolution of 0.5. Published literature and CellMarker (http://bio-bigdata.hrbmu.edu.cn/CellMarker/) were used for cell type annotation. Subclustering of cells with CD79A and CD79B expression was performed in the same way with a resolution of 0.1. Log-normalized expression values of selected genes were compared according to the cell types and pathology results using Seurat.

*Cell ratio analysis*

Cell ratio analysis was conducted to determine the proportion of each cell cluster between the tumor group and the control group (non-tumor). Initially, the proportion of each cell type was calculated by dividing the number of cells of that type by the total number of cells in each sample. Subsequently, a comparison was made between between the tumor samples and the control samples in terms of the proportions of cell types or subtypes.

*Copy number variation (CNV) estimation*

We estimated the initial CNVs for each cell cluster by sorting the analyzed genes based on their chromosomal location. This was done using a moving average of 100 analyzed genes and the equation, as previously reported(6). Next, we applied a re-standardiztion process to the gene expression of each cell cluster, restricting the relative expression values to the range of -1 to 1. Lastly, the CNV score for each cell cluster was obtained by calculating the quadratic sum of the expression values(7).

*Gene expression profiling interactive analysis (GEPIA)*

We employed GEPIA2 (http://gepia2.cancer-pku.cn) to assess the impact of specific genes or gene signatures in our study(8). We utilized the Kaplan-Meier method to determine the disease-free survival (DFS) of chosen genes or gene signatures among with patients with iCCA, employing a 50% (Median) cutoff for both low and high expression group.We employed the Log-rank Mantel-Cox test to identify any significant statistical differences.

*Single-cell trajectory analysis*

We utilized Monocle2 (version 2.24.0) to reconstruct the developmental trajectory of cells expressing CD79A (9). Seurat was used to identify the top 50 differentially expressed genes (DEGs) among subclusters and sort cells based on pseudotime. After the first round of ‘orderCells’, we employed marker genes specific to naive B cells to define the root state argument and subsequently reran the ‘orderCells’ function. We employed ‘DDRTree’ to reduce dimensionality and used ‘plot_cell_trajectory’ to visualize the minimum spanning tree of cells. We calculated DEGs using ‘differentialGeneTest’ in Monocle2 and plotted a heatmap based on significant values (q value < 10^-30^).

*Cell-cell communication analysis*

Based on ligand-receptor (L-R) interactions, CellChat (version 1.4.0) was employed to infer and visualize cell-cell communication between GrB^+^B cells and other cell types(10). Briefly, single-cell RNA-seq data from the control and tumor groups were separately imported and analyzed. Next, we examined the disparities in L-R interactions and signaling pathways between the two groups, with a particular focus on GrB^+^B cells as the receiving cells.

*Functional enrichment analysis*

Highly variable features of GrB^+^B cells in both the control and tumor groups were identified, separately. Subseqwuently, gene ontology (GO) functional enrichment analysis was performed using the database for annotation, visualization, and integrated discovery (DAVID; Version 6.8) (https://david.ncifcrf.gov/, accessed on 8 September 2022). A bar plot was used to display the top 10 analyzed values of biological process (BP) terms. Subsequently, DEGs in B cell subsets between the tumor and control groups were identified. The volcano plot was used to visualize the upregulated and downregulated genes. The gene symbols were subsequently converted to entrezIDs using ‘org.Hs.eg.db’. Functional enrichment analysis was conducted using ‘enrichGO’ within ‘clusterProfiler’.

*Single-cell regulatory network inference and clustering (SCENIC)*

SCENIC (version 1.3.1) was utilized to predict the activity and specificity of transcription factors (TF) based on the single-cell RNA-seq data in this study(11). To accomplish this, the single-cell gene expression profile was used as input to obtain modules of TF-gene co-expression through GENIE3. Subsequently, RcisTarget was employed to refine those modules by selecting the genes that possessed the corresponding TF binding motif in order to construct regulons. Finally, AUCell was employed to assign scores each cell by evaluating the activity and specificity of the regulons, thereby providing insight into TF that may drive the gene network.

*Patients and samples*

Liver transplant recipients (LTR) who had pathologically confirmed iCCA, as well as patients with benign hepatic disease, were included in this study conducted between March 2017 and May 2022. The inclusion criteria for the patients were the presence of a single nodule (≤ 4.5 cm) or two nodules, both measuring ≤ 3 cm without macrovascular invasion or extrahepatic spread, including lymph node metastasis, as determined by imaging or significant elevation of CA199 before surgery. The follow-up period for all participants exceeded 6 months. Notably, the patients with iCCA did not undergo any pre-surgery therapy. CD20^+^B cells were isolated from normal hepatic and iCCA samples, while CD4^+^T cells were obtained from peripheral blood samples of the control group. Tumor and non-tumor tissues from iCCA samples were collected and prepared for immunohistochemistry staining.

*Immunohistochemistry staining*

Immunohistochemistry was conducted using the PV-6000 One-step plus®Poly-HRP Anti-Mouse/Rabbit IgG Detection System (GBI, Bothell, WA, USA), following the manufacturer’s instructions. The samples were treated with primary antibodies against GrB (#TA505931, ZSGB-BIO, Beijing, China), CD20 (#TA800385, ZSGB-BIO, Beijing, China), CD3 (#TA506064, ZSGB-BIO, Beijing, China), CXCL12 (#ab25117, Abcam, Cambridge, UK)，MIF (#ab187064, Abcam, Cambridge, UK), and polyperoxidase anti-mouse/rabbit IgG (#PV-6000, ZSGB-BIO, Beijing, China), respectively. Reactivity was visualized using DAB reagent sets. A negative control was included by substituting the primary antibody with immunoglobulin G.

*Cell preparation*

Tumor-infiltrating lymphocytes (TIL) were obtained from tumors by tissue digestion. Briefly, fresh hepatic tissues were cut into small pieces of approximately 1 mm^3^, digested using various enzymes (Sigma-Aldrich, St. Louis, USA) including 0.002% DNA enzyme (#D4545), 0.05% collagenase (#C6885), and 0.01% hyaluronidase (#H1115000), and filtered. TIL and peripheral blood mononuclear cells (PBMC) were isolated using ficoll density gradient centrifugation.

CD20^+^B cells were purified from TIL (purity ≥ 98%) using a positive selection strategy of magnetic cell separation (CD20 MicroBeads, human, Miltenyi Biotec, Germany). CD4^+^T cells were purified from PBMC (purity ≥ 98%). All experiments were performed using mycoplasma-free cells.

*Functional assay of granzyme B^+^B cells*

A functional assay of granzyme B^+^B cells was conducted to assess the suppressive function of granzyme B^+^B cells, as previously reported(12). Briefly, we stimulated purified CD20^+^B cells with IgG+IgM (5.4 μg/ml, # 309-295-0640, Jackson ImmunoResearch Laboratories, PA, USA), CpG ODN 2006 (9.6 μg/ml, # tlrl-2006-5, InvivoGen, CA, USA), and IL-21 (50 ng/ml, # SRP3087, Sigma-Aldrich, MO, USA). After a 4-day incubation, CFSE-labeled CD4^+^T cells were stimulated with or without pre-activated B cells using the T cell activator (Dynabeads human T activator CD3/CD28, #11163D, Gibco, NY, USA) at a ratio of 1:1 (T cells: B cells). T cell proliferation was quantified using flow cytometry.

*Antibodies (Abs) and flow cytometric measurement*

For the detection of GrB, the following anti-human Abs were used: FITC-Zombie (#423111, Biolegend, CA, USA), PE-GrB (#AB_10372671, eBioscience, CA, USA), APC-fire750-CD69 (#985206 , Biolegend, CA, USA), and PE-Cy7-CD20 (#980208, Biolegend, CA, USA). TIL were harvested and initially stained with surface Abs. Subsequently, fixation/permeabilization was performed using Cytofix/Cytoperm kit (#426803, BD Biosciences, CA, USA), followed by intracellular staining. Flow cytometry analysis was carried out using NovoCyte D2060R instrument (ACEA Biosciences Inc, CA, USA), and data analysis was performed using NovoEXpress software (San Diego, CA, USA). Surface staining alone was employed for BCR and IL21R detection. The following Abs were used: FITC-IgE (#982902), FITC-IgM (#314506), FITC-IgA (#333512), PE-IgG (#410708), PE-IgD (#307804), PE-IL-21R (#131906), and PE-Cy7-CD20 (#560735) from BD Biosciences. Cells analysis was conducted on the DxFLEX instrument (Beckman Coulter, FL, USA) and data were processed using CytExpert software.

*Polymerase chain reaction (PCR) and Western blot*

PCR and Western blot assays were employed to evaluate the production of GrB, as previously reported(13). Briefly, CD20^+^B cells were isolated and incubated with the aforementioned stimuli for approximately 20 hours. For PCR analysis, total RNA was extracted using Trizol (#R0011, Beyotime, Jiangsu, China), and cDNA synthesis was performed using the PrimeScript RT reagent Kit with gDNA Eraser (#RR047, Takara, Tokyo, Japan). The housekeeping gene RPL32 was used as an internal control. The resulting cDNA products were run on a 2% agarose gel after reverse transcription. All PCR assays were conducted on an ABI 7500 Fast Real-Time PCR System (Applied Biosystems, CA, USA). In Western blot analysis, primary Abs were added to samples, followed by a washing step and incubation with the secondary Ab conjugated to HRP for 1 hour. The Ab against β-Actin was purchased from Santa Cruz (#PA1-16889, CA, USA), and the Ab against GrB was obtained from Abcam (#ab32111, Cambridge, UK). The secondary Abs used were anti-rabbit IgG from Santa Cruz and anti-mouse IgG from Solarbio (#06-371, Beijing, China).

*Statistical analysis*

Data were analyzed using SPSS 19.0 computer software (IBM Corp., Armonk, NY, USA). GraphPad Prism 5 software (GraphPad Software Inc., La Jolla, CA, USA) was utilized for generating the figures. Values were expressed as mean ± standard deviation. The normal distribution of continuous variables were assessed using the Kolmogorov–Smirnov test. For quantitative variables, the independent samples t-test was employed. The Wilcoxon ranksum test was selected due to non-normal distribution. The Chi-square or Fisher’s exact test was used to compare nominal variables. All statistical tests were two-sided permutation tests. Kaplan-Meier method was used for survival analysis of the patients. A *P*-value < 0.05 was considered statistically significant.

**References**

1. MacParland SA, Liu JC, Ma XZ, Innes BT, Bartczak AM, Gage BK, et al. Single cell RNA sequencing of human liver reveals distinct intrahepatic macrophage populations. Nat Commun. 2018;9(1):4383.
2. Massalha H, Bahar Halpern K, Abu-Gazala S, Jana T, Massasa EE, Moor AE, et al. A single cell atlas of the human liver tumor microenvironment. Mol Syst Biol. 2020;16(12):e9682.
3. Zhang M, Yang H, Wan L, Wang Z, Wang H, Ge C, et al. Single-cell transcriptomic architecture and intercellular crosstalk of human intrahepatic cholangiocarcinoma. J Hepatol. 2020;73(5):1118-30.
4. Ma L, Hernandez MO, Zhao Y, Mehta M, Tran B, Kelly M, et al. Tumor Cell Biodiversity Drives Microenvironmental Reprogramming in Liver Cancer. Cancer Cell. 2019;36(4):418-30 e6.
5. McGinnis CS, Murrow LM, Gartner ZJ. DoubletFinder: Doublet Detection in Single-Cell RNA Sequencing Data Using Artificial Nearest Neighbors. Cell Syst. 2019;8(4):329-37 e4.
6. Patel AP, Tirosh I, Trombetta JJ, Shalek AK, Gillespie SM, Wakimoto H, et al. Single-cell RNA-seq highlights intratumoral heterogeneity in primary glioblastoma. Science. 2014;344(6190):1396-401.
7. Peng J, Sun BF, Chen CY, Zhou JY, Chen YS, Chen H, et al. Single-cell RNA-seq highlights intra-tumoral heterogeneity and malignant progression in pancreatic ductal adenocarcinoma. Cell Res. 2019;29(9):725-38.
8. Tang Z, Kang B, Li C, Chen T, Zhang Z. GEPIA2: an enhanced web server for large-scale expression profiling and interactive analysis. Nucleic Acids Res. 2019;47(W1):W556-W60.
9. Trapnell C, Cacchiarelli D, Grimsby J, Pokharel P, Li S, Morse M, et al. The dynamics and regulators of cell fate decisions are revealed by pseudotemporal ordering of single cells. Nat Biotechnol. 2014;32(4):381-6.
10. Jin S, Guerrero-Juarez CF, Zhang L, Chang I, Ramos R, Kuan CH, et al. Inference and analysis of cell-cell communication using CellChat. Nat Commun. 2021;12(1):1088.
11. Aibar S, Gonzalez-Blas CB, Moerman T, Huynh-Thu VA, Imrichova H, Hulselmans G, et al. SCENIC: single-cell regulatory network inference and clustering. Nat Methods. 2017;14(11):1083-6.
12. Xu WL, Wang RL, Liu Z, Wu Q, Li XL, He Q, et al. Granzyme B-Producing B Cells Function as a Feedback Loop for T Helper Cells in Liver Transplant Recipients with Acute Rejection. Inflammation. 2021;44(6):2270-8.
13. Li H, Li XL, Cao S, Jia YN, Wang RL, Xu WL, et al. Decreased granzyme B(+)CD19(+)B cells are associated with tumor progression following liver transplantation. Am J Cancer Res. 2021;11(9):4485-99.

**Supporting figures and figure legends**


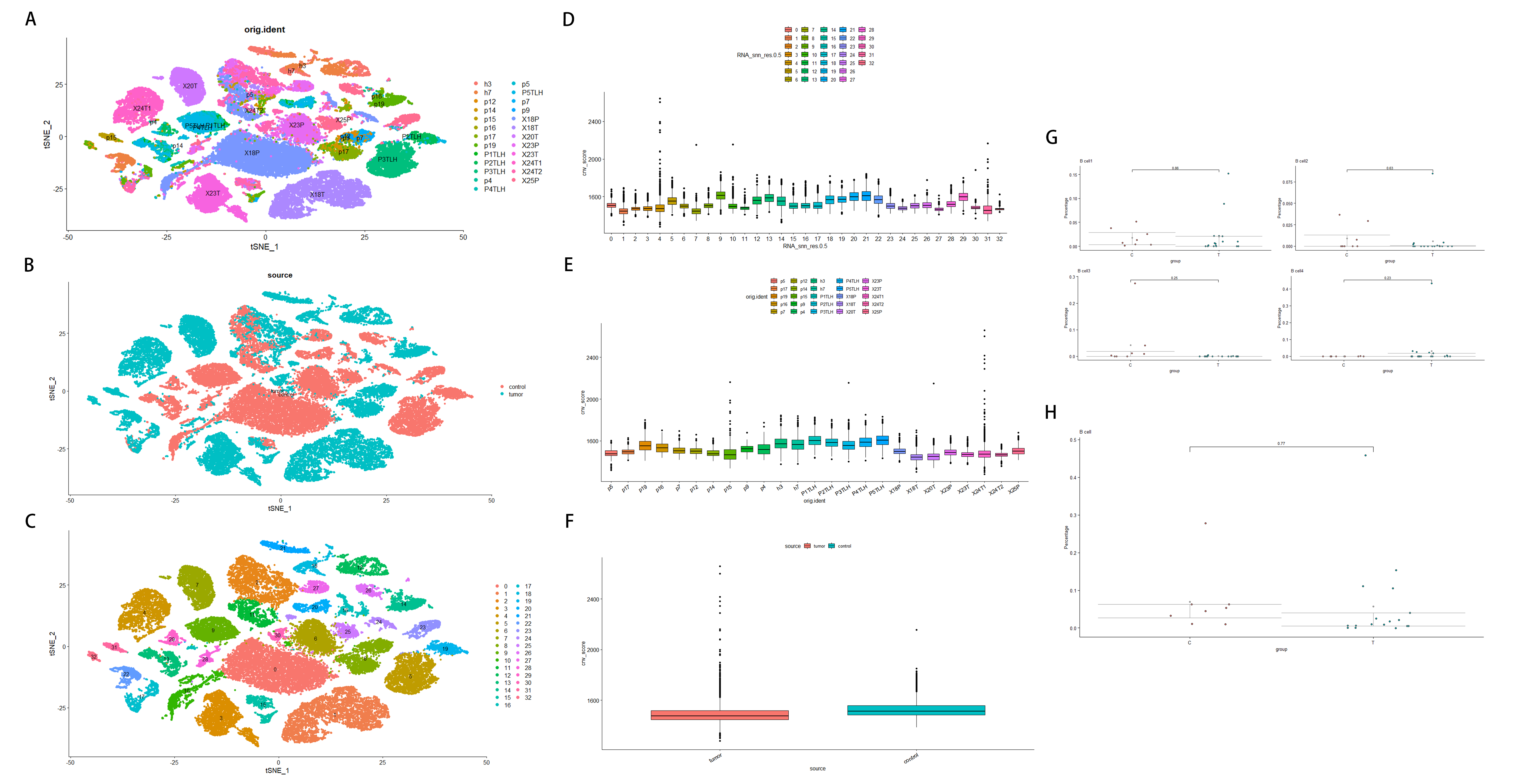


Figure S1.Copy number variation (CNV) and transcriptome landscape of single cells in iCCA and control livers

A The t-distributed stochastic neighbor embedding (t-SNE) plot demonstrating the origin of the cells detected in hepatic samples. B The t-SNE plot demonstrating the malignant and non-malignant cells in hepatic samples. C The t-SNE plot demonstrating the 33 clusters identified. D The box line plot showing distributions of CNV scores among different clusters. E The box line plot showing distributions of CNV scores among different hepatic samples. F The box line plot showing distributions of CNV scores between the tumor and control groups. Comparison of proportions of B cell clusters in part (G) and in total (H) between the control and tumor groups.

Figure S2. Characterization of GrB^+^B cells identified in B cell clusters

A The t-distributed stochastic neighbor embedding (t-SNE) plot demonstrating the origin of the B cells detected in hepatic samples. B The t-SNE plot demonstrating the 9 subclusters identified. C Heatmap showing expression levels of specific markers in each B cell subcluster. D Dot plots demonstrating the expression of marker genes for the B cell subtypes. E Potential developmental trajectory of B cells inferred by Monocle 2 based on gene expression. F Clustered heatmap showing the dynamic changes of gene expression with pseudotime. G Proportions of the B cell subtypes in the total B cell population in hepatic samples.


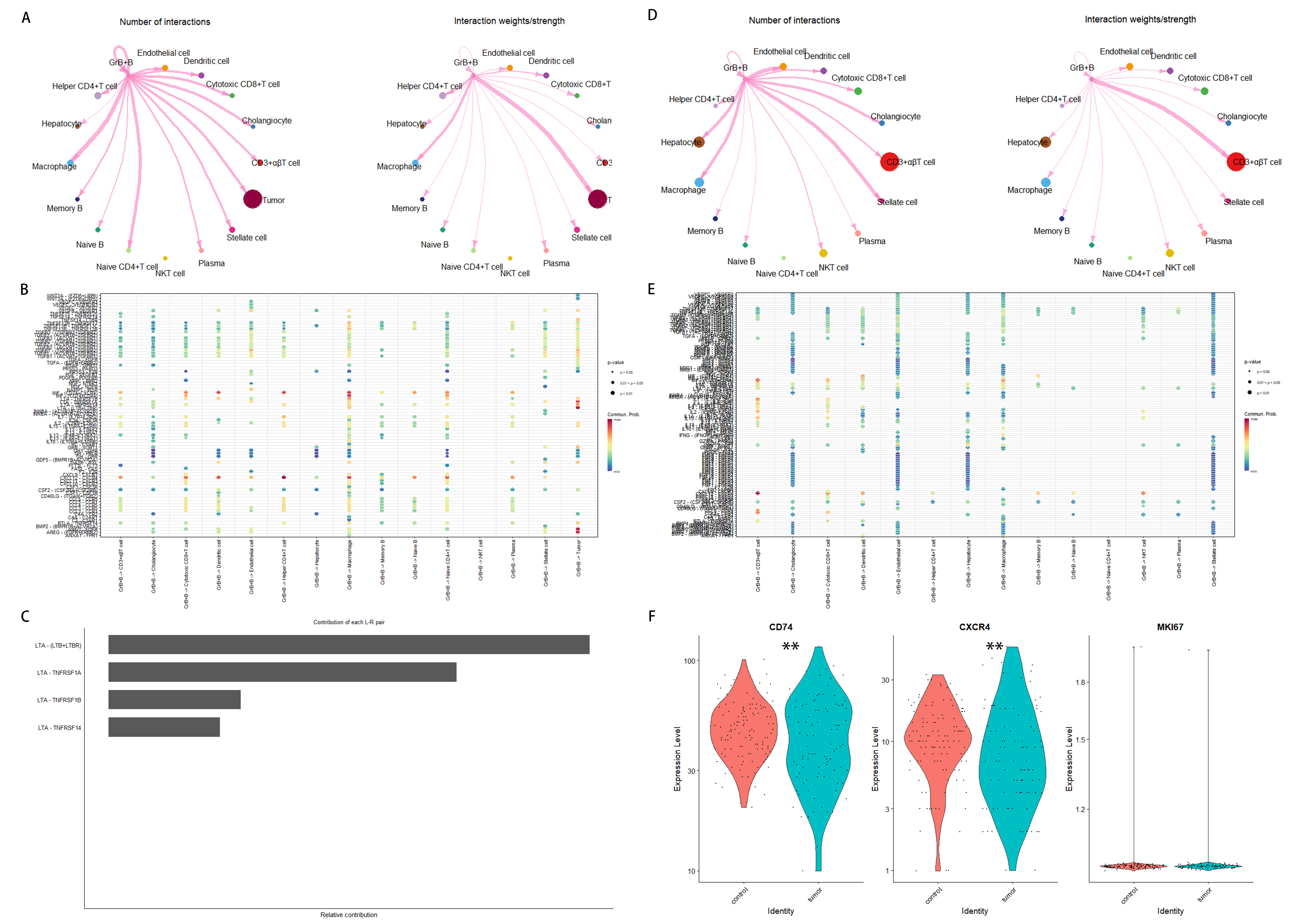


Figure S3. Enriched GrB^+^B cells in patients with iCCA result from chemotaxis of tumor cells

A Circle plots showing number of interactions and interaction weights/strength between GrB^+^B cells as a ligand and receptor cells including CD3^+^αβT cells, cholangiocytes, cytotoxic CD8^+^T cells, dendritic cells, endothelial cells, helper CD4^+^T cells, hepatocytes, macrophages, memory B cells, naive B cells, naive CD4^+^T cells, NKT cells, plasma cells, stellate cells, and tumor cells in tumor samples. Dot size indicates the number of each cell type. The lines connect to the cell types that express the cognate receptors. The line thickness is proportional to the number of ligand-receptor pairs. B Dot plot displaying the ligand-receptor (L-R) pairs in tumor samples, which contribute to the signaling from GrB^+^B cells to receptor cells. Dot color reflects communication probabilities and dot size represents computed *P-*values. Empty space means the communication probability is zero. C Relative contribution of each L-R pair to the overall LT signaling network. D Circle plots showing number of interactions and interaction weights/strength between GrB^+^B cells as a ligand and receptor cells including CD3^+^αβT cells, cholangiocytes, cytotoxic CD8^+^T cells, dendritic cells, endothelial cells, helper CD4^+^T cells, hepatocytes, macrophages, memory B cells, naive B cells, naive CD4^+^T cells, NKT cells, plasma cells, and stellate cells in control samples. E Dot plot displaying the L-R pairs in control samples with GrB^+^B cells as a ligand. F Violin plots showing the expression distributions of CD74, CXCR4, and MKI67 in GrB^+^B cells from the tumor and control samples involved in the corresponding signaling network with comparison of log-normalized gene expression values, respectively. ***CD74, *P* = 1.0155e-08; ***CXCR4, *P* = 5.774879e-06; MKI67, n.a


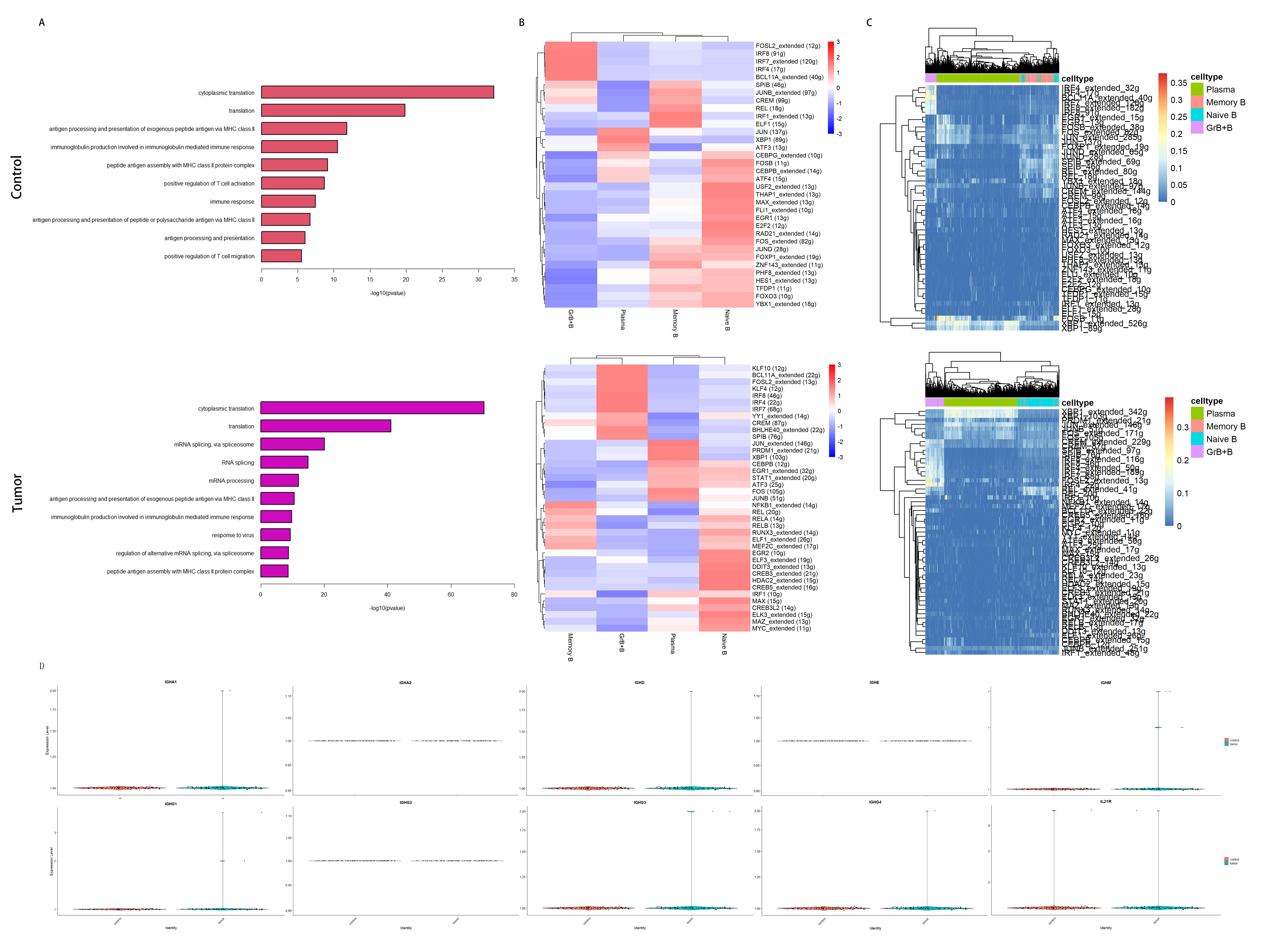


Figure S4. The changes in GrB^+^B cells’ functional states between the tumor and control groups

A Top 10 biological pathways enriched for highly variable features identified in GrB^+^B cells from the control (up) and tumor (down) samples, respectively. Bars represent numbers of highly variable features in each GO term. B Heatmaps of the area under the curve (AUC) scores of regulon specificity of B cell subsets from the control (up) and tumor (down) samples, respectively as estimated using SCENIC. C Heatmaps of the AUC scores of regulon activity of B cell subsets from the control (up) and tumor (down) samples, respectively. D Violin plots showing the expression distributions of IGHA1, IGHA2, IGHD, IGHE, IGHM, IGHG1, IGHG2, IGHG3, IGHG4 and IL21R in B cells from the tumor and control samples with comparison of log-normalized gene expression values, respectively.


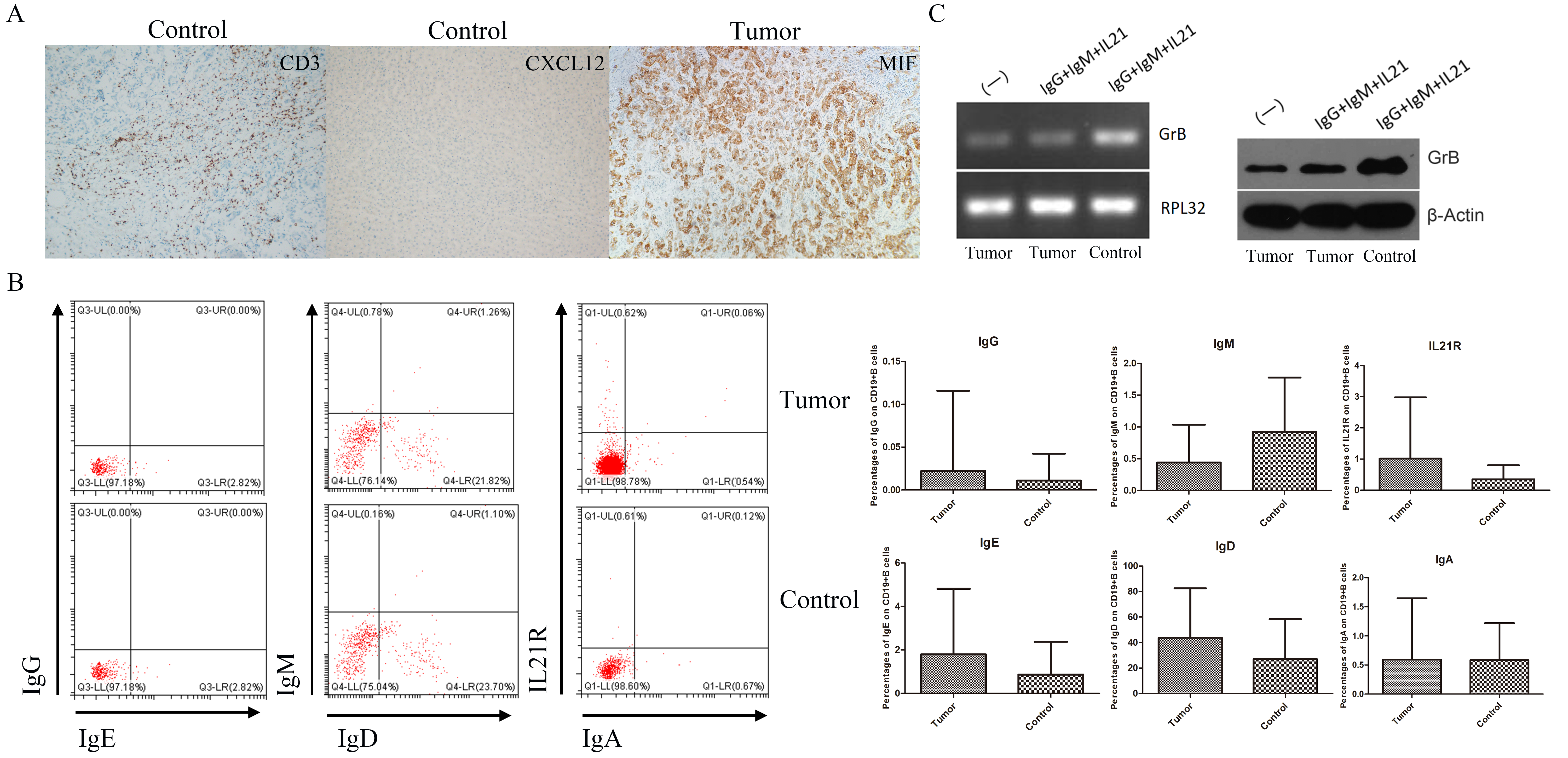


Figure S5. Increased but compromised GrB^+^B cells validated in a clinic cohort

A Representative immunohistochemistry images of the CD3 (left) and CXCL12 (middle) expressions in benign hepatic tissues and the MIF (right) expression in iCCA cells, respectively. B Representative flow cytometry dot plots and comparison of the BCR and IL21R expressions in resting CD20^+^B cells from tumor (upper row, n=35) and control (lower row, n=11) samples, respectively. C Granzyme B expression in CD20^+^B cells from the tumor and control groups detected by polymerase chain reaction (left, n=3) and Western blotting (right, n=3) under various conditions, respectively. CD20^+^B cells were stimulated in the presence or absence of IgG+IgM (5.4 μg/ml) and IL-21 (50 ng/ml). iCCA, intrahepatic cholangiocarcinoma
